# Effect of blood source on vector competence of *Culex pipiens* biotypes for Usutu virus

**DOI:** 10.1186/s13071-021-04686-6

**Published:** 2021-04-08

**Authors:** Sandra R. Abbo, Tessa M. Visser, Constantianus J. M. Koenraadt, Gorben P. Pijlman, Haidong Wang

**Affiliations:** 1 Laboratory of Virology, Wageningen University & Research, Droevendaalsesteeg 1, 6708 PB Wageningen, The Netherlands; 2 Laboratory of Entomology, Wageningen University & Research, Droevendaalsesteeg 1, 6708 PB Wageningen, The Netherlands

**Keywords:** Blood source, Arbovirus, Usutu virus, Transmission, *Culex pipiens*, Mosquito, Vector competence

## Abstract

**Background:**

Infectious blood meal experiments have been frequently performed with different virus-vector combinations to assess the transmission potential of arthropod-borne (arbo)viruses. A wide variety of host blood sources have been used to deliver arboviruses to their arthropod vectors in laboratory studies. The type of blood used during vector competence experiments does not always reflect the blood from the viremic vertebrate hosts in the field, but little is known about the effect of blood source on the experimental outcome of vector competence studies. Here we investigated the effect of avian versus human blood on the infection and transmission rates of the zoonotic Usutu virus (USUV) in its primary mosquito vector *Culex pipiens*.

**Methods:**

*Cx. pipiens* biotypes (*pipiens* and *molestus*) were orally infected with USUV through infectious blood meals containing either chicken or human whole blood. The USUV infection and transmission rates were determined by checking mosquito bodies and saliva for USUV presence after 14 days of incubation at 28 °C. In addition, viral titers were determined for USUV-positive mosquito bodies and saliva.

**Results:**

Human and chicken blood lead to similar USUV transmission rates for *Cx. pipiens* biotype *pipiens* (18% and 15%, respectively), while human blood moderately but not significantly increased the transmission rate (30%) compared to chicken blood (17%) for biotype *molestus*. USUV infection rates with human blood were consistently higher in both *Cx. pipiens* biotypes compared to chicken blood. In virus-positive mosquitoes, USUV body and saliva titers did not differ between mosquitoes taking either human or chicken blood. Importantly, biotype *molestus* had much lower USUV saliva titers compared to biotype *pipiens*, regardless of which blood was offered.

**Conclusions:**

Infection of mosquitoes with human blood led to higher USUV infection rates as compared to chicken blood. However, the blood source had no effect on the vector competence for USUV. Interestingly, biotype *molestus* is less likely to transmit USUV compared to biotype *pipiens* due to very low virus titers in the saliva.

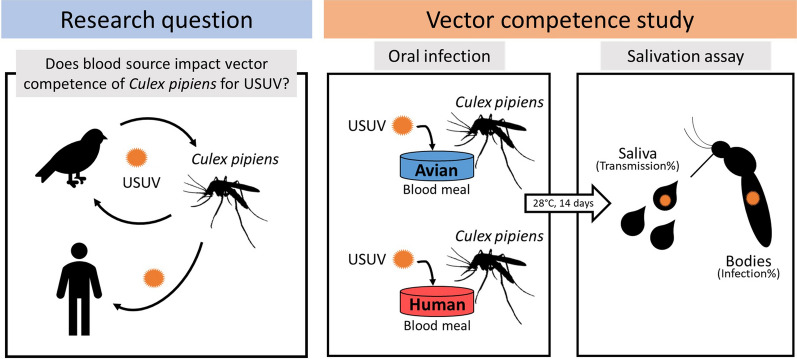

## Background

Arthropod-borne (arbo)viruses can cause severe disease outbreaks in animals and humans. The spread of arboviruses is mainly determined by the presence of competent vectors, often mosquitoes. A female mosquito can acquire a virus during blood feeding on a viremic vertebrate host. After the extrinsic incubation period, the infected mosquito can transmit the virus via the mosquito saliva by feeding on a next host [1, 2]. The ability of a mosquito species to transmit a certain virus is defined as the vector competence [2, 3]. Vector competence has been investigated for many virus-vector combinations [4–6], and helps to assess the risk of viral emergence and spread.

During vector competence studies, infectious blood meals are commonly offered via artificial feeding systems, where a mixture of virus and blood is contained in either a reservoir covered with a membrane (natural or Parafilm) or is supplied via droplets or a pledget of cotton wool [7]. The type of blood used for artificial feeding differs per study but often resembles the blood from vertebrate hosts involved in the virus transmission cycles. However, the actual experimental setups can also be constrained by the availability of certain blood sources. Therefore, the blood used during vector competence experiments does not always resemble the blood from the viremic hosts in the field. Little is known about the impact of blood source on vector competence [3].

The zoonotic Usutu virus (USUV; family *Flaviviridae*, genus *Flavivirus*) is currently circulating in Europe and is drawing increasing attention due to its substantial mortality in avian species and the potential to cause neurological disease in humans [8]. Vector competence studies are therefore important to assess the risk of USUV outbreaks in Europe and beyond. In the field, USUV is primarily transmitted between avian reservoir hosts and mosquitoes [9–12]. Humans and other mammals can be infected with USUV via mosquito bites; however, they are considered dead-end hosts due to low levels of viremia [13]. Hence, the use of avian blood in the infectious blood meal experiments with USUV is therefore preferred [14]. Nonetheless, infectious blood meals containing human blood [15], swine blood [16], sheep blood [17], horse blood [18], bovine blood [19] or rabbit blood [20] have been used to assess the vector competence of local mosquito species for USUV. The different outcomes of these studies are often attributed to virus strains or mosquito lines, while the use of different sources of blood during artificial feeding is often not discussed.

Here we investigated the effect of blood source on the transmission of USUV by the common house mosquito *Culex pipiens*, which is the primary vector for USUV in Europe [9]. *Cx. pipiens* consists of two biotypes among which biotype *pipiens* prefers to feed on birds, whereas biotype *molestus* preferentially feeds on mammals including humans [21]. We compared the USUV infection and transmission rates of both *Cx. pipiens* biotypes after ingestion of an infectious blood meal containing either chicken or human whole blood. The viral titers in the body and saliva of the USUV-positive mosquitoes were also compared.

## Methods

### Mosquitoes, cells and viruses

Previously established *Cx. pipiens* biotype *pipiens* and biotype *molestus* colonies from the Netherlands [22] were reared separately as described earlier [22]. Mosquitoes were maintained at 23 °C with a 16:8 light/dark cycle and relative humidity of 60%.

African green monkey kidney Vero E6 cells were routinely cultured in Dulbecco’s modified Eagle medium (DMEM; Gibco, Carlsbad, CA, USA) with 10% fetal bovine serum (FBS; Gibco), penicillin (100 U/ml; Sigma-Aldrich, St. Louis, MO, USA) and streptomycin (100 μg/ml; Sigma-Aldrich) (P/S) at 37 °C with 5% CO_2_. Preceding virus infections, Vero cells were seeded in HEPES-buffered DMEM medium (Gibco) supplemented with 10% FBS and P/S. When Vero cells were incubated with mosquito body lysate or saliva, the HEPES-buffered DMEM medium was additionally supplemented with Gentamicin (50 μg/ml; Gibco) and Fungizone (2.5 μg/ml of amphotericin B and 2.1 μg/ml of sodium deoxycholate; Gibco). This medium will hereafter be referred to as DMEM HEPES complete.

Passage 5 and 6 virus stocks of USUV, the Netherlands 2016 (GenBank accession no. MH891847.1; EVAg Ref-SKU 011 V-02153; obtained from Erasmus Medical Center, Rotterdam, the Netherlands), were grown on Vero cells. Viral titers, expressed as 50% tissue culture infectious dose per milliliter (TCID_50_/ml), were determined by end-point dilution assays (EPDAs) on Vero cells using 60-well MicroWell plates (Nunc, Roskilde, Denmark).

### Infectious blood meal

Before the infectious blood meal, 3–18-day-old mosquitoes were starved for 1 day. Infectious blood meal experiments were conducted in the biosafety level 3 laboratory of Wageningen University & Research. Mosquitoes were orally exposed to chicken whole blood (Kemperkip, Uden, the Netherlands) or human whole blood (Sanquin Blood Supply Foundation, Nijmegen, the Netherlands) containing 10^7^ TCID_50_/ml of USUV. Mosquitoes were fed in a dark room for 1 h using a Hemotek PS5 feeder (Discovery Workshops, Lancashire, United Kingdom). Infectious chicken blood was provided during four (*molestus*) and three (*pipiens*) independent experiments, whereas both biotypes were exposed to infectious human blood in three independent experiments. After the blood meal, mosquitoes were immobilized using 100% CO_2_, and the fully engorged females were selected. A small number of females was stored at −80 °C in SafeSeal micro tubes (Sarstedt, Nümbrecht, Germany) containing 0.5 mm zirconium oxide beads (Next Advance, Averill Park, NY, USA) to measure the viral titer in the mosquito body immediately after engorgement. All remaining females were incubated at 28 °C for 14 days. A 6% glucose solution was provided as food source.

### Salivation assay

Fourteen days post-infection, mosquito saliva was collected by forced salivation as described earlier [23]. Mosquitoes were first immobilized using 100% CO_2_, and their legs and wings were removed. To collect mosquito saliva, the mosquito proboscis was inserted in a 200 µl pipette tip holding 5 µl of a 1:1 mixture of FBS and 50% sugar in autoclaved tap water. After 45 min, the samples containing mosquito saliva were mixed with 55 μl DMEM HEPES complete and stored at −80 °C. The mosquito bodies were collected in SafeSeal micro tubes (Sarstedt) containing 0.5 mm zirconium oxide beads (Next Advance) and also stored at −80 °C.

### Infectivity assay

Mosquito body samples were taken from −80 °C and directly homogenized in a Bullet Blender Storm (Next Advance) at maximum speed for 2 min. Next, homogenates were spun down in an Eppendorf 5424 centrifuge at 14,500 rpm for 1 min. One hundred microliters of DMEM HEPES complete was then added to each sample. The homogenates in medium were blended again at maximum speed for 2 min, and centrifuged at 14,500 rpm for 2 min. From each body or saliva sample, 30 μl was added to one well of a 96-well plate containing Vero cells in DMEM HEPES complete. After 2 h at 37 °C, the medium of the cells was removed and replenished with fresh DMEM HEPES complete. After 6 days of incubation at 37 °C, the cells were inspected for cytopathic effect (CPE), and each well was scored virus-positive or virus-negative. For a subset of the results, these scores were also confirmed by reverse transcriptase PCR on total RNA isolated from Vero cells using primers against the region coding for USUV non-structural protein 5 as previously described [24]. The infection and transmission rates were then calculated by expressing the number of virus-positive mosquito bodies or saliva as a percentage of the total number of mosquitoes analyzed. Viral titers of mosquito bodies and saliva were measured using EPDAs on Vero cells. After 6 days at 37 °C, wells were considered virus-positive or virus-negative based on CPE.

### Statistical analysis

Fisher’s exact test was used to compare the infection and transmission rates between human and chicken infectious blood meals. The Shapiro–Wilk test was used to check the normality of log-transformed viral titer data sets. Then the Mann–Whitney *U* test was used to compare the mean viral titers between two log-transformed data sets. Statistical tests were performed in GraphPad Prism 5.

## Results

### The effect of blood source on USUV infection and transmission rates in *Cx. pipiens*

**Fig. 1:**
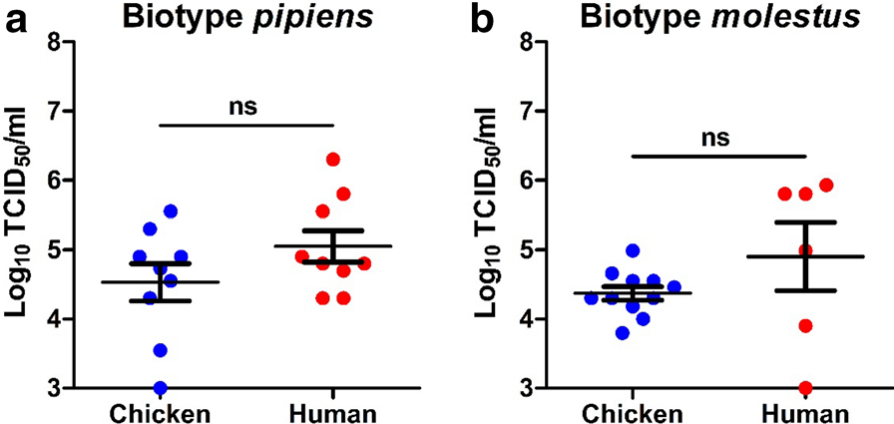
USUV uptake in *Cx. pipiens* mosquitoes immediately after engorgement of an infectious blood meal. The blood meal consisted of either chicken or human blood. Virus titers were determined by EPDAs for (**a**) biotype *pipiens* and (**b**) biotype *molestus*. Data points represent individual mosquitoes orally exposed to USUV. Lines among the dots indicate the mean viral titers. Error bars show the standard error of the mean (SEM). A non-significant difference is indicated by ns between two data sets (*p* > 0.05; Mann–Whitney *U* test)

**Fig. 2:**
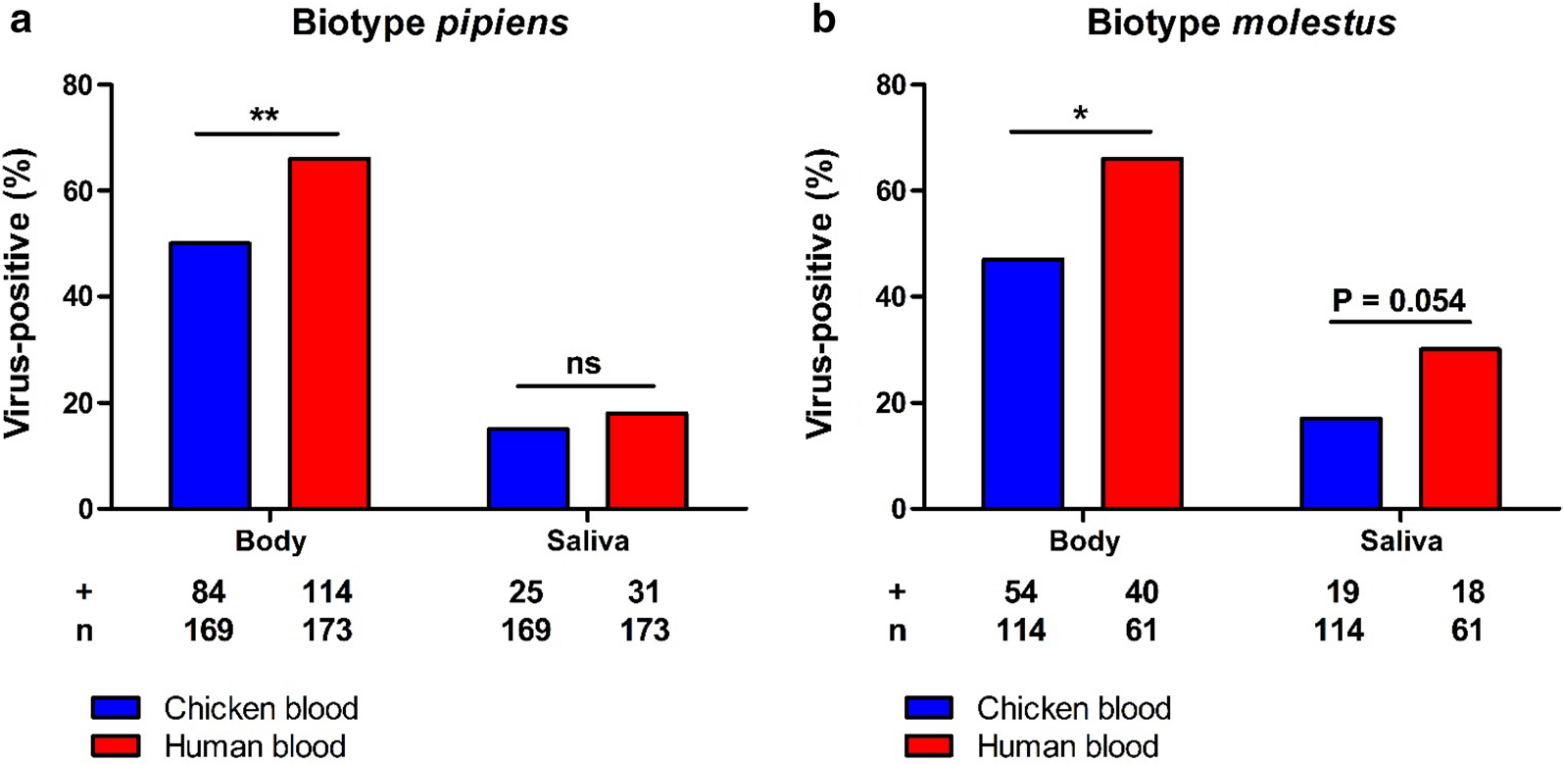
Infection and transmission of USUV after oral exposure of *Cx. pipiens* to infectious chicken or human blood. **a** *Cx. pipiens pipiens* and **b** *Cx. pipiens molestus* were incubated at 28 °C, and analyzed for infectious virus at 14 days post-blood meal. The number of virus-positive mosquito bodies or saliva samples (indicated by +) is expressed as a percentage of the total number of mosquitoes tested (indicated by n). Asterisks (*, **) indicate a significant *p* value of < 0.05 and < 0.01, respectively, while ns represents a non-significant difference (Fisher’s exact test)

The effect of blood source on USUV infection and transmission was investigated for both biotypes. A selection of females was used to determine the viral titers in the mosquito bodies immediately after oral ingestion. For both *Cx. pipiens* biotypes, the mean viral titers in the mosquito bodies right after the oral feeding were similar between the two types of infectious blood meal [(*p* = 0.351; Fig. 1a) and (*p* = 0.267; Fig. 1b)]. All other fully engorged females were maintained at 28 °C for 14 days. Out of the biotype *pipiens* mosquitoes fed with infectious chicken blood, 50% showed virus-positive bodies after 14 days, whereas human blood resulted in a higher body infection rate of 66% (*p* = 0.003; Fig. 2a). The percentage of mosquitoes with USUV-positive saliva was similar among the biotype *pipiens* mosquitoes exposed to either chicken or human infectious blood (15% and 18%, respectively; *p* = 0.467; Fig. 2a). For biotype *molestus*, the avian infectious blood infected 47% of the engorged mosquitoes, whereas the human infectious blood infected a significantly higher percentage of the mosquitoes (66%; *p* = 0.026; Fig. 2b). The USUV transmission rate was somewhat higher for biotype *molestus* provided with human blood (30%) compared to chicken blood (17%), but the significance was marginal (*p* = 0.054; Fig. 2b). Altogether, these results indicate that blood source did not significantly impact the vector competence of *Cx. pipiens* biotypes for USUV.

### The effect of blood source on USUV titers in bodies and saliva of *Cx. pipiens*

**Fig. 3:**
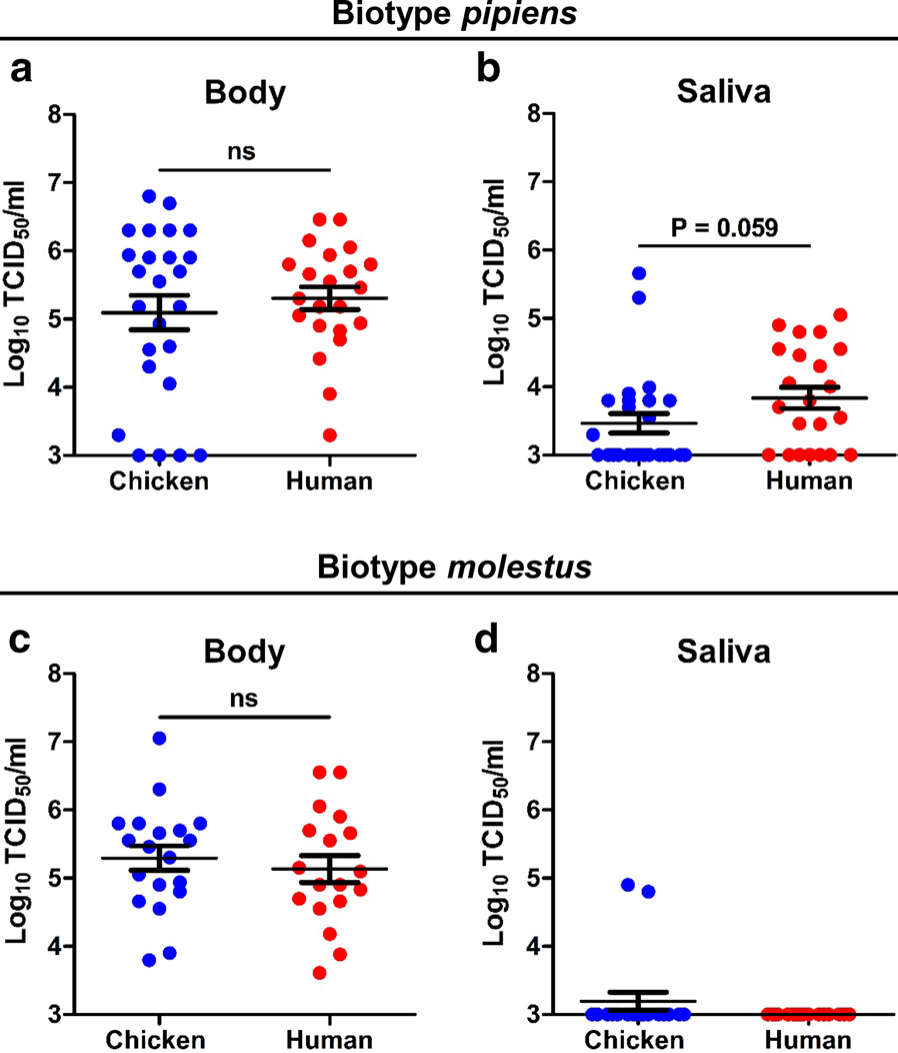
USUV titers in bodies and saliva of *Cx. pipiens* after oral exposure to infectious chicken or human blood. **a**, **b** *Cx. pipiens pipiens* and **c**, **d** *Cx. pipiens molestus* were incubated at 28 °C, and virus titers were determined 14 days post-infection. Data points represent individual mosquitoes exposed to USUV. Lines among the dots indicate the mean viral titers. Error bars show the standard error of the mean (SEM). A non-significant difference is indicated by ns (*p* > 0.05; Mann–Whitney *U* test)

We next looked at the USUV titers in *Cx. pipiens* bodies and saliva at 14 days after the oral ingestion. Although human blood resulted in a higher USUV infection rate for *Cx. pipiens* biotype *pipiens*, the mean viral body titers were not significantly different between mosquitoes that took up either a chicken or a human infectious blood meal (10^5.1^ and 10^5.3^ TCID_50_/ml, respectively; *p* = 0.898; Fig. 3a). The mean USUV saliva titers of *Cx. pipiens* biotype *pipiens* were slightly higher when mosquitoes were offered a human blood meal (10^3.8^ TCID_50_/ml) compared to a chicken blood meal (10^3.5^ TCID_50_/ml), although the difference is only marginally significant (*p* = 0.059; Fig. 3b). For *Cx. pipiens* biotype *molestus*, we found that the mean viral body titers showed similar values of 10^5.3^ and 10^5.1^ for chicken and human blood, respectively (*p* = 0.574; Fig. 3c). Surprisingly, viral saliva titers for biotype *molestus* were all below the detection limit of our EPDA (10^3^ TCID_50_/ml) except two “outliers” (Fig. 3d). Therefore, no differences can be observed between human and chicken blood. Collectively, these results show that blood source did not significantly affect the viral titers in *Cx. pipiens* biotypes after oral exposure to USUV.

## Discussion

Infectious blood meal experiments are frequently performed on mosquitoes and other vector species to investigate their vector competence for arboviruses. Here we provided *Cx. pipiens*, the primary vector for USUV, with infectious blood meals containing either chicken or human whole blood, and investigated the effect of blood source on the infection and transmission of USUV. We found that both types of blood lead to comparable vector competence of the two *Cx. pipiens* biotypes for USUV. Other sources of mammalian blood, e.g. sheep [17] and rabbit [20], have been used to investigate the vector competence of *Culex* spp*.* for the prototype strain of USUV (SAAR-1776) under laboratory conditions. In line with our observations, these studies also confirmed the vector competence of *Culex* spp. for USUV. Interestingly, another study, in which equine blood was used, reported limited transmission of USUV (SAAR-1776) in two *Cx. pipiens* lines [18]. It remains unclear whether the equine blood plays a role in contributing to the low viral transmission. Parallel oral infections using equine blood and another type of blood in the infectious blood meal experiments might be helpful to rule out any effect of blood source on the measured vector competence.

In our study, the source of blood used for oral feeding did not affect the virus titer in bodies of USUV-infected mosquitoes. A similar result was observed for *Culex tarsalis* mosquitoes infected with western equine encephalomyelitis virus (WEEV; family *Togaviridae*, genus *Alphavirus*), where mean titers of WEEV in mosquito bodies did not differ significantly when mosquitoes were provided with either chicken or rabbit blood [25]. Moreover, WEEV infection rates did not vary significantly among mosquitoes fed with chicken or rabbit blood [25]. In our study, however, we found that human blood consistently generated a higher USUV infection rate among the tested *Cx. pipiens* biotypes compared to chicken blood. Further studies about the impact of different blood sources on the measured infection and transmission rates for other virus-vector combinations could help to further clarify the effect of blood source on the outcome of artificial feeding experiments, and will hopefully allow for a better assessment of the competence of vector species for arboviruses.

It is not entirely understood how host blood could impact arbovirus infection in the arthropod vector, but certain host-derived factors have a role to play [7]. For example, species-specific serum proteases, of which the presence depends on the blood source, can cleave the outer capsid protein VP2 of African horse sickness virus, thereby resulting in enhanced infectivity in *Culicoides* midges [26]. In addition, a recent study has shown that different types of ingested blood result in diverse bacterial compositions in the midgut of vector mosquito *Aedes aegypti* [27]. The gut bacterial microbiome of mosquitoes has proven to be a potent modulator of arbovirus infection [28–30], and it is therefore possible that the host blood can influence arbovirus infection through modulation of the mosquito bacterial microbiome. Future studies on how host blood reshapes the microbiome in the mosquito midgut and potentially alters the outcome of arbovirus infection are therefore needed.

Finally, we found that the viral saliva titers of biotype *molestus* at 14 days post-infection were much lower compared to the viral saliva titers of biotype *pipiens*, regardless of which blood was offered. These low viral saliva titers of biotype *molestus* may indicate a lower transmission potential for USUV compared to biotype *pipiens*. Since the mean viral titer in biotype *molestus* bodies was around 10^5^ TCID_50_/ml, which is also comparable to that of biotype *pipiens*, the low viral saliva titers in biotype *molestus* are unlikely due to insufficient viral replication in the mosquito bodies. In addition to that, both *Cx. pipiens* biotypes were maintained under the same conditions, thus the difference in USUV titers in the saliva between the two *Cx. pipiens* biotypes is very likely attributed to genetic factors determining the characteristics of mosquito midguts or salivary glands. This finding could suggest that biotype *molestus*, which preferentially feeds on mammals including humans, is a less efficient vector for USUV compared to biotype *pipiens*.

## Conclusions

This study shows for the first time the impact of host blood source on the infection and transmission rates of USUV in *Cx. pipiens* mosquitoes. The USUV transmission rate and viral accumulation in the body and saliva of *Cx. pipiens* mosquitoes were not significantly affected by the type of blood used during artificial feeding. The USUV infection rate, however, was found to be significantly higher for mosquitoes provided with infectious human blood compared to mosquitoes that received infectious chicken blood. In addition, *Cx. pipiens* biotype *molestus* is less likely to transmit USUV as compared to biotype *pipiens* due to very low virus titers in the saliva.

## Data Availability

All data generated or analyzed during this study are included in this published article.
